# Supplementary material for: The ion channel CALHM6 controls bacterial infection‐induced cellular cross‐talk at the immunological synapse
Source: EMBO J. 2023 Mar 2;42(7):e111450. doi: 10.15252/embj.2022111450 (PMC10068325; doi:10.15252/embj.2022111450)
Supplement: Supplementary file 1 — Expanded View Figures PDF [file EMBJ-42-e111450-s004.pdf]

Expanded View Figures

Figure EV1. Generation and characterisation of *Calhm6*$^{-/-}$ mice.

A A diagram of the gene cassette used to generate *Calhm6*$^{-/-}$ mice. In PGK-Cre strain, Cre is driven by the early acting PGK-1 promoter.

B Immunoblot of CALHM6 in BMDM from WT and *Calhm6*$^{-/-}$ mice stimulated with IFN-γ (10 ng/ml) for 24 h (one representative experiment of two is shown, WT mice = 2, *Calhm6*$^{-/-}$ mice = 2).

C–E Cells from spleen (C and D) or thymus (E) of WT and *Calhm6*$^{-/-}$ mice were isolated and analysed by flow cytometry after staining with antibodies against lymphoid (C and E) or innate cell (D) surface markers. Represented are the % total live cells (C and E) or % CD19^{-} cells (D) (WT mice $n = 19$ *Calhm6*$^{-/-}$ mice $n = 16$).

F, G CALHM6 is dispensable for the instruction of adaptive memory responses to *Listeria monocytogenes* (pooled results from three independent experiments, WT mice = 19, *Calhm6*$^{-/-}$ mice = 16, multiple *t*-test). (F) WT and *Calhm6*$^{-/-}$ mice were injected i.p. with low dose (3×10^4 CFU) of *L. monocytogenes*. On day 3 spleens were harvested from sacrificed mice, mechanically disrupted, and plated on antibiotic-free BHI agar plates to determine CFU as a measure of primary response to infection. (G) IFN-γ in serum from mice in (F) measured by ELISA (Representative experiment is shown) (one representative experiment of four, WT mice = 17, *Calhm6*$^{-/-}$ mice = 20, Mann–Whitney test).

H–J Experimental scheme for memory response: WT and *Calhm6*$^{-/-}$ mice were injected with 3×10^5 CFU of *L. monocytogenes* i.p. and then rested. After 6 weeks these mice were challenged with 3×10^6 CFU. CFU/g (I) in spleen and (J) liver 3 days after challenge were measured by plating spleens and livers mechanically dispersed on antibiotic-free BHI agar plate (pooled results from four independent experiments, spleen: WT mice = 17, *Calhm6*$^{-/-}$ mice = 17, liver: WT mice = 21, *Calhm6*$^{-/-}$ mice = 18, Mann–Whitney test).

K, L IFN-γ but not granzyme B production is impaired in NK cells of *Calhm6*$^{-/-}$ mice. WT and *Calhm6*$^{-/-}$ mice were injected i.p. with Poly(I:C) (100 μg/mouse) or PBS. After 3 h splenocytes were collected and incubated for an additional 4 h with Brefeldin A before intracellular staining for IFN-γ and analysis by flow cytometry. (K) Representative contour plots and (L) pooled percentage of IFN-γ^{+} and/or Granzyme B^{+} $CD3^{-}NK1.1^{+}$ in Poly(I:C) injected WT and *Calhm6*$^{-/-}$ mice (pooled results from two independent experiments, WT mice = 10, *Calhm6*$^{-/-}$ mice = 10, one-way ANOVA with multiple comparisons).

Data information: *$P < 0.05$, **$P < 0.01$, ***$P < 0.001$. Error bars represent SD.
Source data are available online for this figure.

© 2023 The Authors

© 2023 The Authors

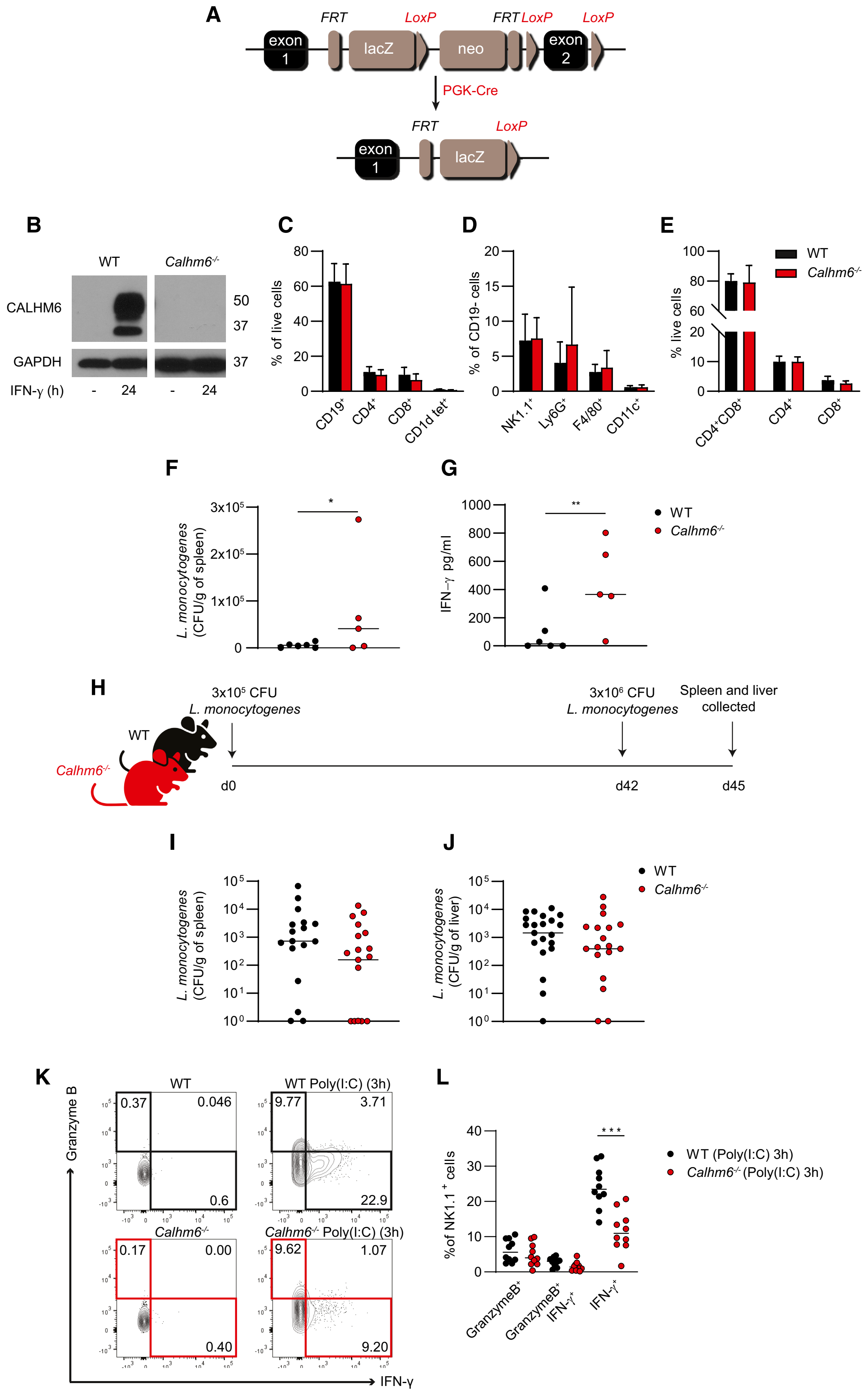

Figure EV1.

 © 2023 The Authors

Figure EV2. *Calhm6$^{-/-}$* macrophages have a normal type I IFN response to Poly(I:C) injection.

A Splenocytes obtained from WT and *Calhm6$^{-/-}$* mice after 3 h Poly(I:C) (200 μg/mouse) or PBS injection were incubated for an additional 1 h with PMA/Ionomycin and then with Brefeldin A for an additional 3 h. Cells were stained for intracellular IFN-γ and analysed by flow cytometry (results from one experiment, Poly(I:C) WT mice = 5, *Calhm6$^{-/-}$* mice = 5, control WT mice = 2, *Calhm6$^{-/-}$* mice = 2).

B–I Experimental scheme: WT and *Calhm6$^{-/-}$* mice were injected with Poly(I:C) (200 μg/mouse) or PBS i.p. Spleens were collected 3 h and 6 h later and F4/80^{+} cells isolated with magnetic beads selection to collect RNA. Serum was collected from the injected mice 1 h or 3 h after Poly(I:C) (200 μg/mouse) injection and cytokines measured using LegendPlex kit. Gene expression of *Ifna4* (C), *Ifnb1* (D), *Ifna14* (E), *Il1r2* (F), *Il12a* (G), *Il12b* (H) and *Calhm6* (I) was assessed by qPCR (WT mice = 5 and 5, *Calhm6$^{-/-}$* mice = 5 and 5, control mice = 1 and 1). Differences in these genes (expect *Calhm6* itself) between WT and *Calhm6$^{-/-}$* were not statistically significant (results from one experiment, Poly(I:C) WT mice = 5, *Calhm6$^{-/-}$* mice = 5, control WT mice = 1, *Calhm6$^{-/-}$* mice = 1).

J Membrane IL-15ra expression on BMDCs after overnight LPS (100 ng/ml) or Poly(I:C) (50 μg/ml) treatment was measured by flow cytometry. MFI levels were normalised to WT set to 1 to allow pooling of independent experiments (pooled results from two independent experiments, WT mice = 6, *Calhm6$^{-/-}$* mice = 6).

K–R Indicated serum cytokine levels from poly(I:C) injected mice were measured using Macrophage LegendPlex kit, except IFNβ which was measured using ELISA (pooled results from two independent experiments, Poly(I:C) WT mice = 8, *Calhm6$^{-/-}$* mice = 8–10, control mice = 4–5). Differences in these serum cytokines between WT and *Calhm6$^{-/-}$* were not statistically significant.

Data information: *$P < 0.05$, ****$P < 0.0001$. Test = one-way ANOVA with multiple comparisons, error bars represent SD.

© 2023 The Authors

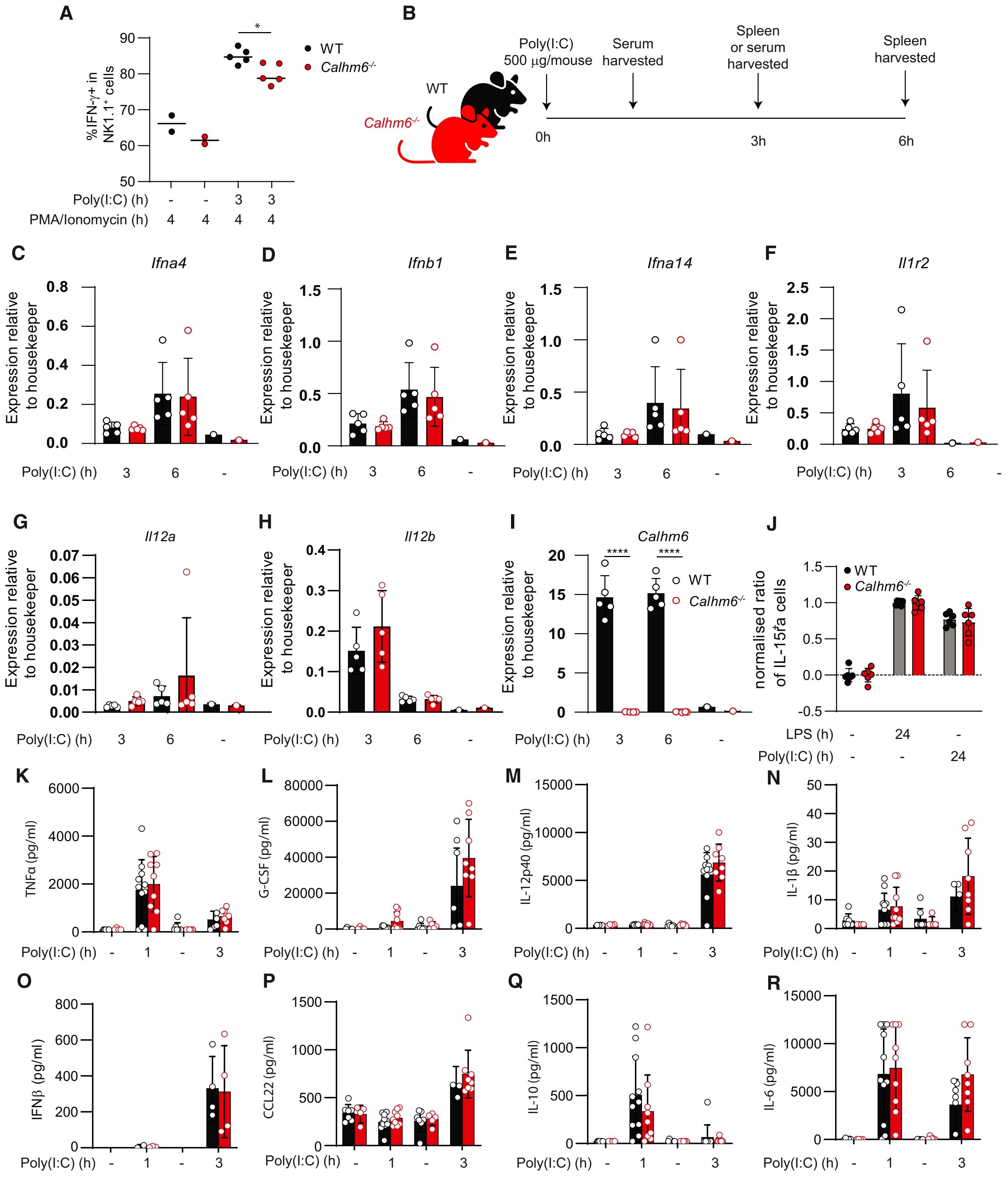

Figure EV2.

 © 2023 The Authors

Figure EV3. CALHM6 on $CD11c^+$ dendritic cells is dispensable for NK cell activation and IFN-γ production.

A Breeding strategy to generate *Itgax*$^{cre/-}$*Calhm6*$^{fl/fl}$ mice. *Calhm6*$^{fl/fl}$ mice were crossed with flippase-expressing mice to excise the LacZ and Neo cassette. The resulting progeny was then crossed with a line expressing a CD11c-specific Cre (*Itgax*$^{cre/-}$) to obtain a CD11c-driven conditional CALHM6 deletion.

B Experimental setup: WT, *Itgax*$^{cre/-}$*Calhm6*$^{fl/fl}$ and *Itgax*$^{cre/-}$ control mice were injected i.p. with Poly(I:C) (200 μg/mouse) or PBS. After 3 h splenocytes were harvested and incubated with Brefeldin A for an additional 4 h, stained for IFN-γ and Granzyme B and analysed by flow cytometry.

C, D Pooled flow cytometry results showing the percentage of IFN-γ^+ (C) or Granzyme B^+ (D) in $NK1.1^+$ cells (pooled results from two independent experiments: WT mice = 7, *Itgax*$^{cre/-}$*Calhm6*$^{fl/fl}$ = 7, *Itgax*$^{cre/-}$ = 3, control = 2 and 2).

E, F WT and *Calhm6*$^{-/-}$ BMDCs (1.5×10^5) or BMDM (1.5×10^5) were co-cultured with 3×10^5 primary WT and *Calhm6*$^{-/-}$ NK cells negatively isolated (enriched) with magnetic beads from murine spleens. Cells were left untreated or stimulated with Poly(I:C) (50 μg/ml) for 24 h. Supernatant was collected, and IFN-γ measured with ELISA (E, pooled results from three independent experiments, WT mice = 7, *Calhm6*$^{-/-}$ mice = 8) (F, pooled results from two independent experiments, WT mice = 5, *Calhm6*$^{-/-}$ mice = 5).

G Co-culture of primary F4/80^+ and $NK1.1^+$ cells isolated from *Rag*$^{-/-}$ and *Rag*$^{-/-}$*Calhm6*$^{-/-}$ mice. Primary F4/80^+ (1.5×10^5) magnetically isolated from mouse spleen were co-cultured with 3×10^5 $NK1.1^+CD3^-$ FACS sorted NK cells as per Fig EV3E (pooled results from two independent experiments, WT mice = 6, *Calhm6*$^{-/-}$ mice = 6).

H Spleen cells (3×10^5) harvested from naïve *Rag*$^{-/-}$ and *Rag*$^{-/-}$*Calhm6*$^{-/-}$ were cultured with or without Poly(I:C) (50 μg/ml) stimulation. After 24 h supernatant was collected, and IFN-γ measured by ELISA (results from one experiment, WT mice = 3, *Calhm6*$^{-/-}$ mice = 3).

Data information: Test = Kruskal–Wallis test, error bars represent SD.

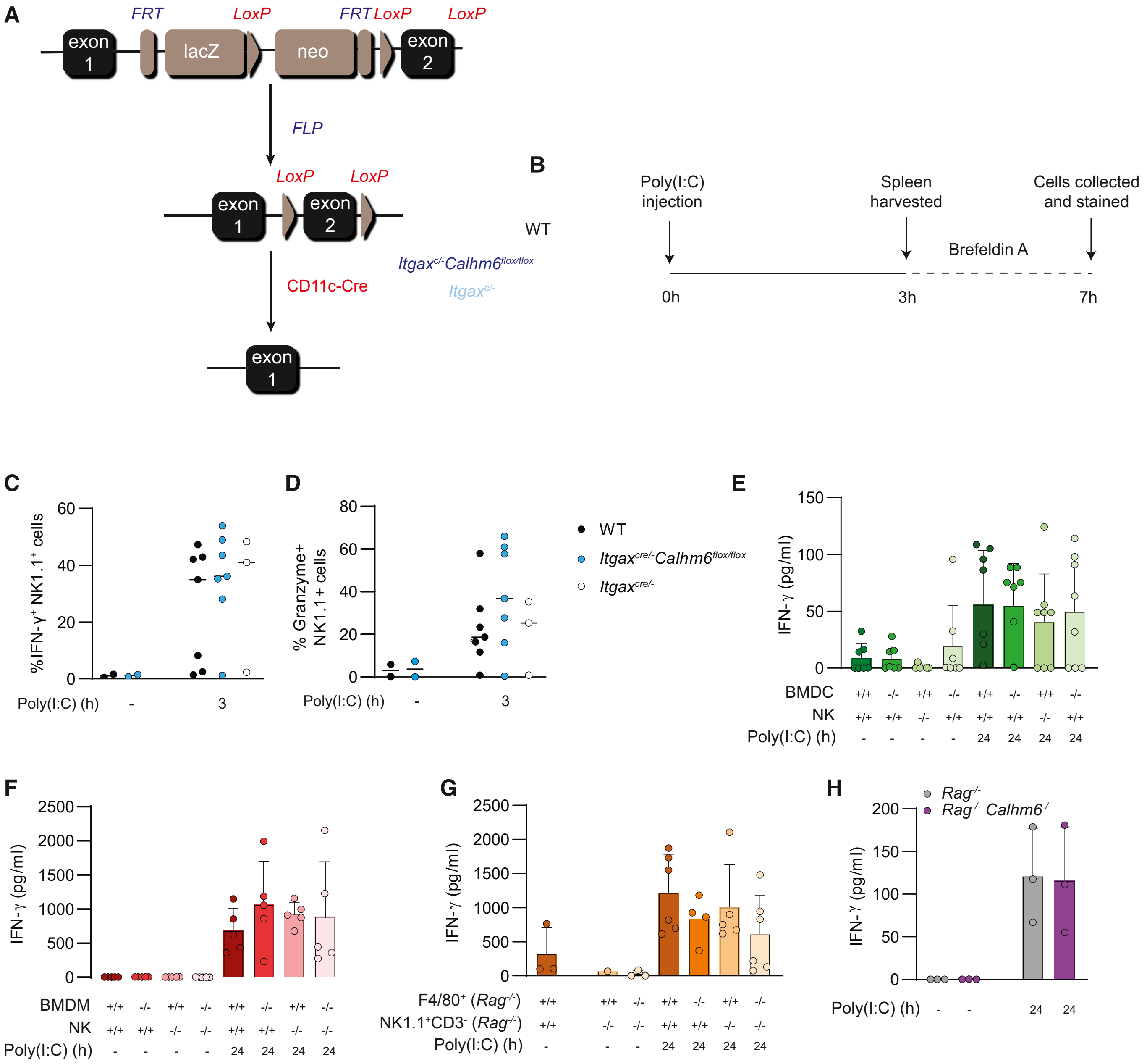

Figure EV3.

© 2023 The Authors

Figure EV4. CALHM6 is recruited to phagocytic synapse but is not required for phagocytosis.

A, B Freshly isolated bone marrow cells were transduced with a retroviral vector expressing CALHM6-FLAG (A) or CALHM6-eGFP (B) and then differentiated into BMDM. BMDM were then stained with phalloidin and DAPI and, in the case of CALHM6-FLAG, fluorescently conjugated αFLAG antibody (representative images are shown).

C, D 3 μM polystyrene beads were added for 0, 10, 20 and 40 min on top of BMDM transduced with CALHM6-eGFP that were pre-treated with IFN-γ (10 ng/ml) and LPS (10 ng/ml) for 6 h. All synaptic interactions are indicated by white arrowheads (representative image in (C), with aggregate result shown in (D) Numbers on top indicate *n* number scored)

E, F PKH26 stained sRBC were added for 0, 5, 10 and 20 min on top of BMDM prepared as in (C). All synaptic interactions are indicated by white arrowheads (representative image in (E), with aggregate result shown in (F) Numbers on top indicate *n* number scored). Images were taken with a 60× objective. *n* of synapses observed for each condition on top of each.

G IgG2a-opsonised sRBC or non-opsonised sRBC were stained with PKH26 and added to WT and *Calhm6*$^{-/-}$ BMDM for 5, 15 and 45 min. At the end of the assay cells were washed with PBS and extracellular sRBCs lysed with RBC lysis buffer, BMDM were fixed with 4% PFA and amount of phagocytosed sRBCs analysed by flow cytometry (one representative experiment of two, WT mice = 2, *Calhm6*$^{-/-}$ mice = 2).

H, I WT and *Calhm6*$^{-/-}$ BMDM were co-cultured with pHrodo Red Zymosan bioparticles (H, pooled results from three independent experiments, WT mice = 3, *Calhm6*$^{-/-}$ mice = 3) or pHrodo Red Dextran 10,000 MW (I, pooled results from three independent experiments, WT mice = 2, *Calhm6*$^{-/-}$ mice = 2). Rate of phagocytosis and endocytosis, were analysed by flow cytometry. MFI in (H) were normalised to maximum phagocytosis set to 100 to allow pooling of independent experiments.

Data information: Test = Two-way ANOVA, error bars represent SD.
Source data are available online for this figure.

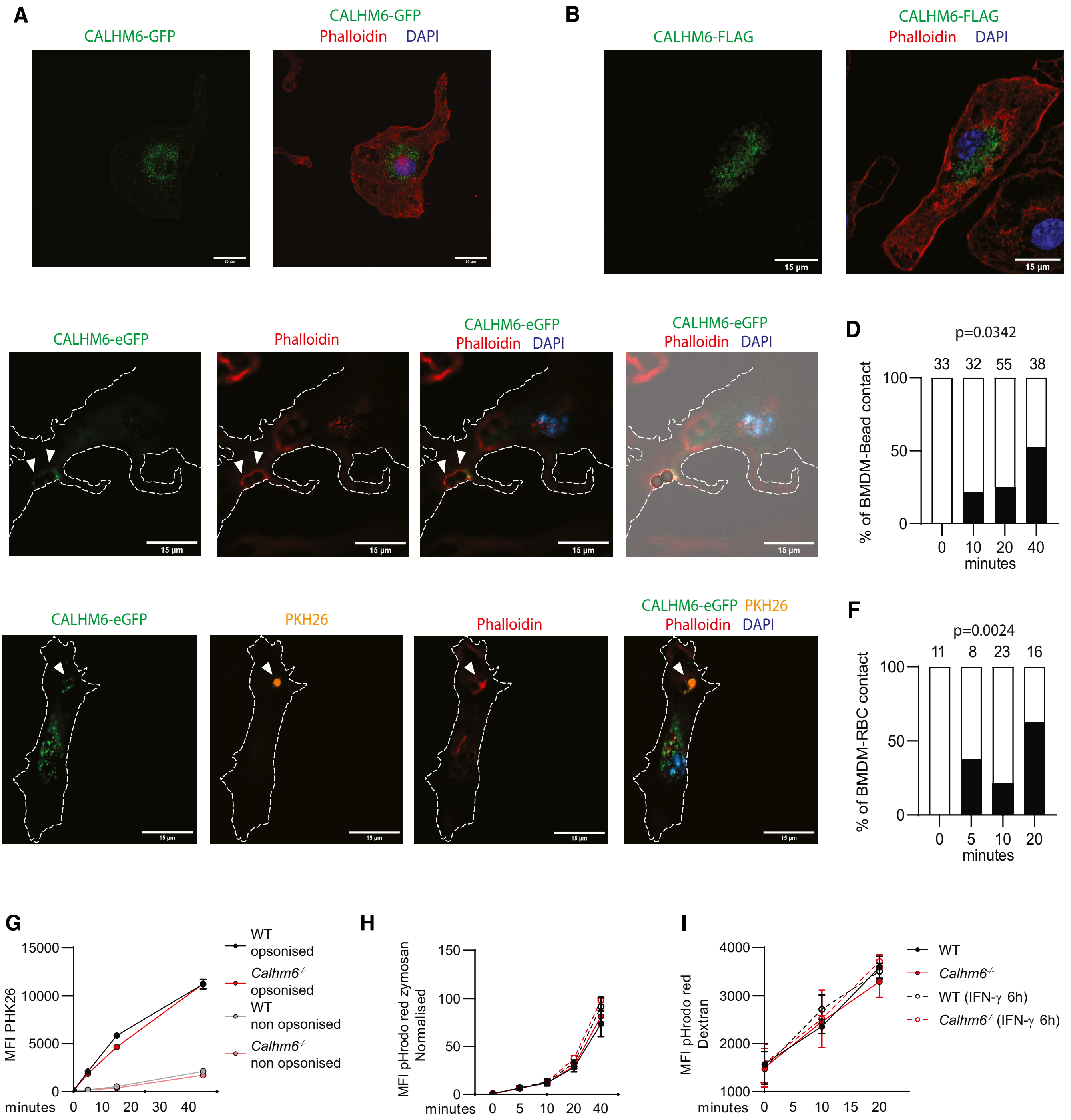

Figure EV4.

 © 2023 The Authors

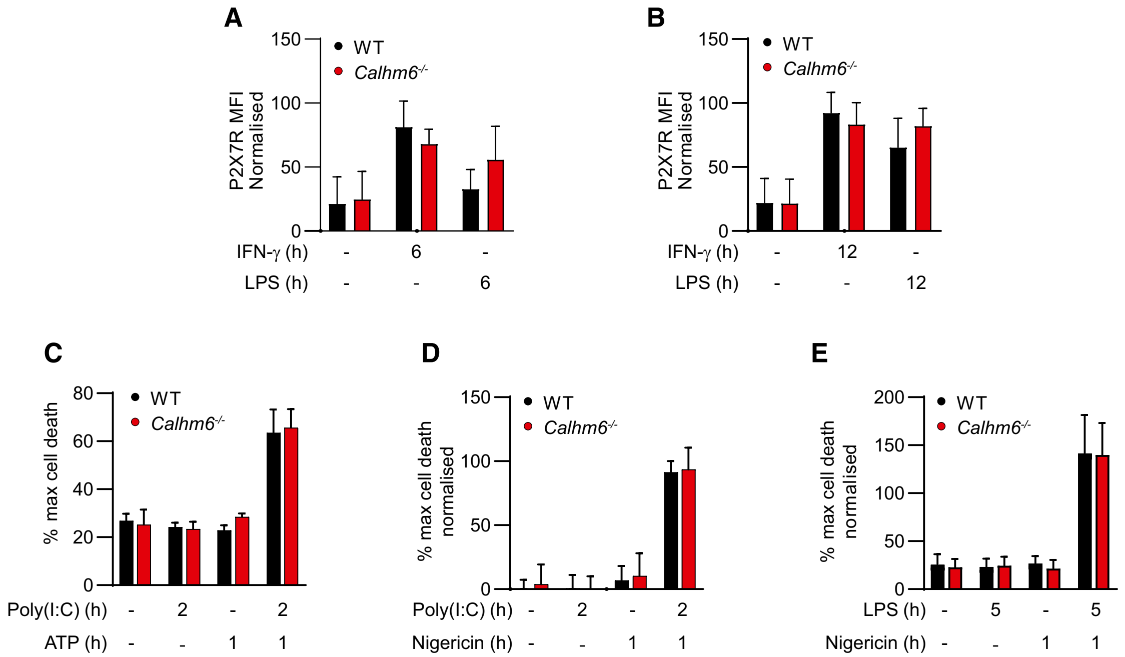

Figure EV5. CALHM6 does not act as a chaperone for ATP receptor P2X7R and is not controlling P2X7R signalling in BMDM.

A, B BMDM were stimulated for 6 h (A) or 12 h (B) with Poly(I:C) (50 µg/ml) or LPS (100 ng/ml) cell surface P2X7R measured by flow cytometry. MFI were normalised to the highest value, considered as 100, to allow pooling of independent experiments (A, pooled results from three independent experiments, WT mice = 3, *Calhm6*$^{-/-}$ mice = 3) (B, pooled results from two independent experiments, WT mice = 2, *Calhm6*$^{-/-}$ mice = 2).

C–E Percentage of cell death measured with LDH assay after inflammasome activation in BMDM: BMDM were treated with Poly(I:C) (50 µg/ml) for 2 h followed by 1 h of ATP (2.5 mM), or nigericin (5 µM) stimulation (C, Representative experiment shown, WT mice = 2, *Calhm6*$^{-/-}$ mice = 2) (D, pooled results from two independent experiments, WT mice = 4, *Calhm6*$^{-/-}$ mice = 4). (E) BMDM were stimulated with LPS (100 ng/ml) for 5 h in total and nigericin (5 µM) was added 1 h before harvest (pooled results from three independent experiments, WT mice = 3, *Calhm6*$^{-/-}$ mice = 3).

Data information: Test = Kruskal–Wallis test with multiple comparisons, error bars represent SD.

© 2023 The Authors
