# Supplementary figures and images for: The ion channel CALHM6 controls bacterial infection‐induced cellular cross‐talk at the immunological synapse
Source: EMBO J. 2023 Mar 2;42(7):e111450. doi: 10.15252/embj.2022111450 (PMC10068325; doi:10.15252/embj.2022111450)

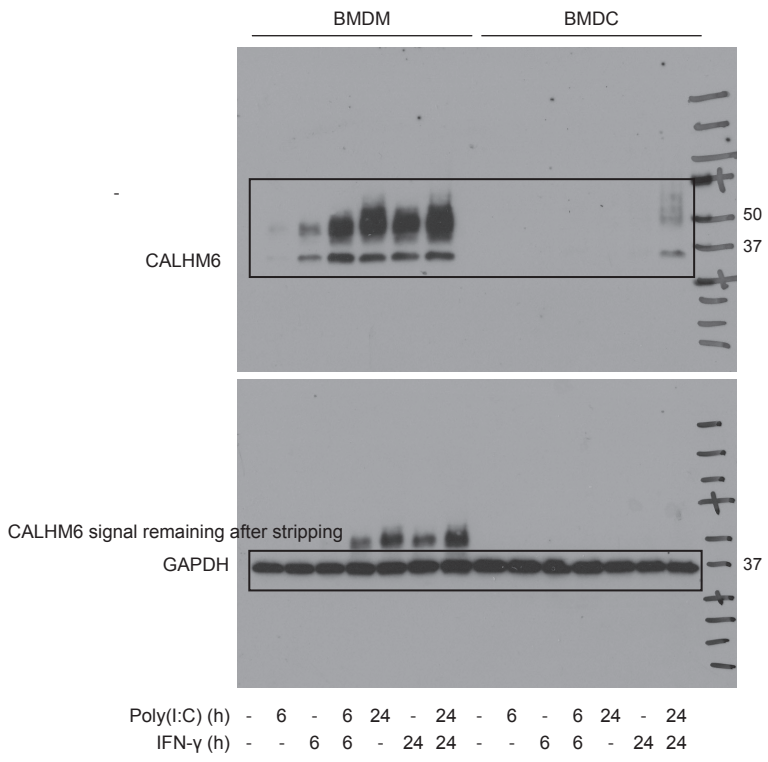

Supplement: Supplementary file 6 — Source Data for Figure 1 [file EMBJ-42-e111450-s010.zip › Source data Figure 1/1B/1B.pdf]

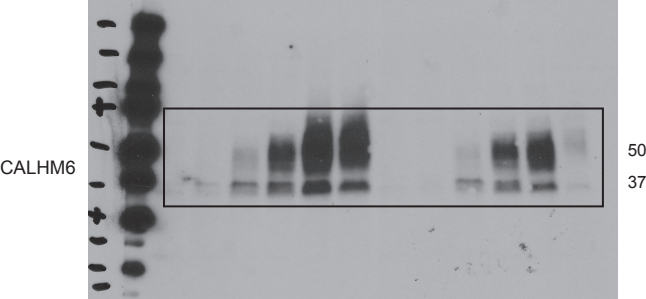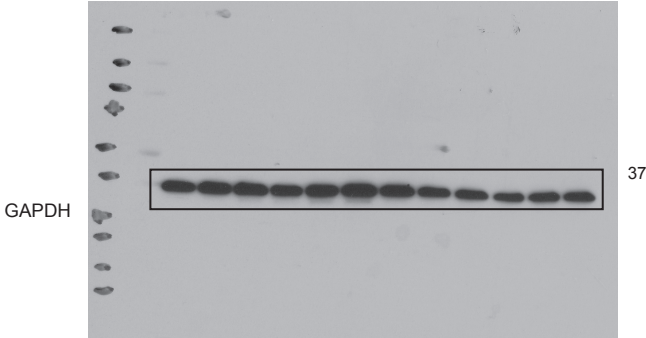

|                   |   |   |   |   |    |    |   |   |   |   |    |    |
|-------------------|---|---|---|---|----|----|---|---|---|---|----|----|
| IFN- $\gamma$ (h) | - | 2 | 4 | 8 | 24 | 48 | - | - | - | - | -  | -  |
| LPS (h)           | - | - | - | - | -  | -  | - | 2 | 4 | 8 | 24 | 48 |

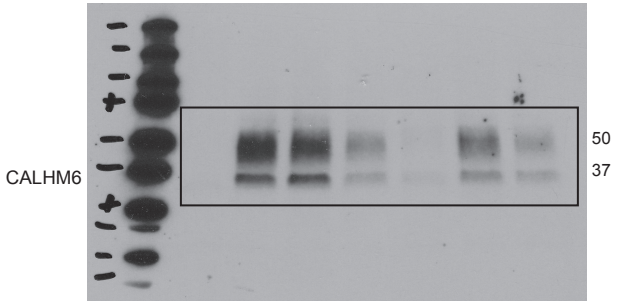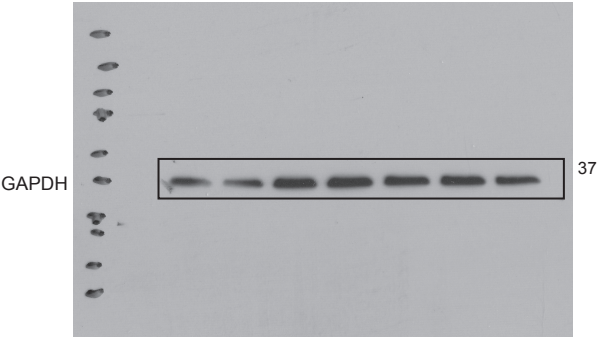

|                   |   |   |   |   |   |   |   |
|-------------------|---|---|---|---|---|---|---|
| LPS (h)           | - | 8 | - | 8 | - | 8 | - |
| IFN- $\gamma$ (h) | - | - | 8 | - | 8 | - | 8 |
| TGF- $\beta$ (h)  | - | - | - | 8 | 8 | 4 | 4 |

Supplement: Supplementary file 6 — Source Data for Figure 1 [file EMBJ-42-e111450-s010.zip › Source data Figure 1/1D&F/1D and F.pdf]

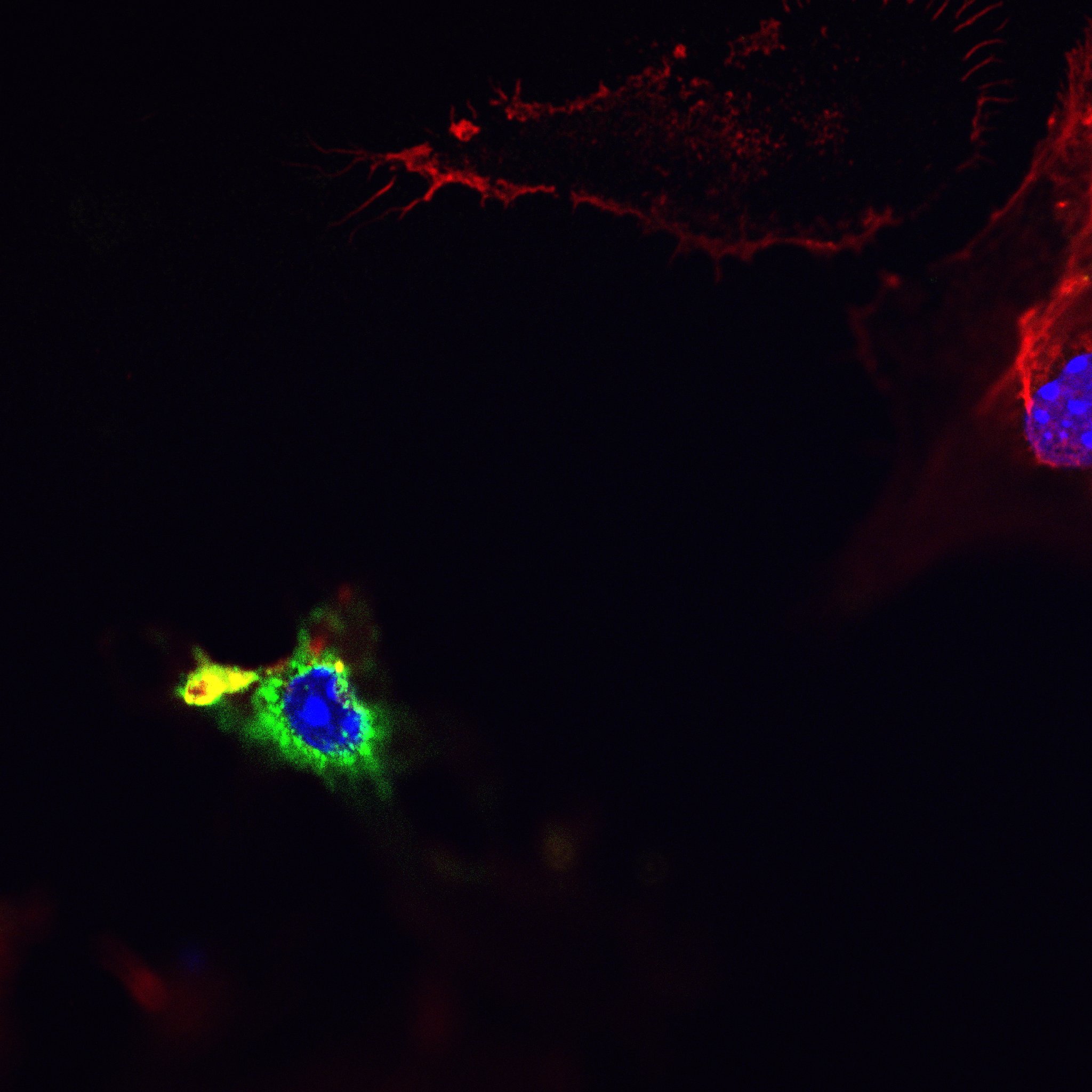

Supplement: Supplementary file 9 — Source Data for Figure 5 [file EMBJ-42-e111450-s005.zip › Source data Figure 5/5F&G/5F all colours.jpg]

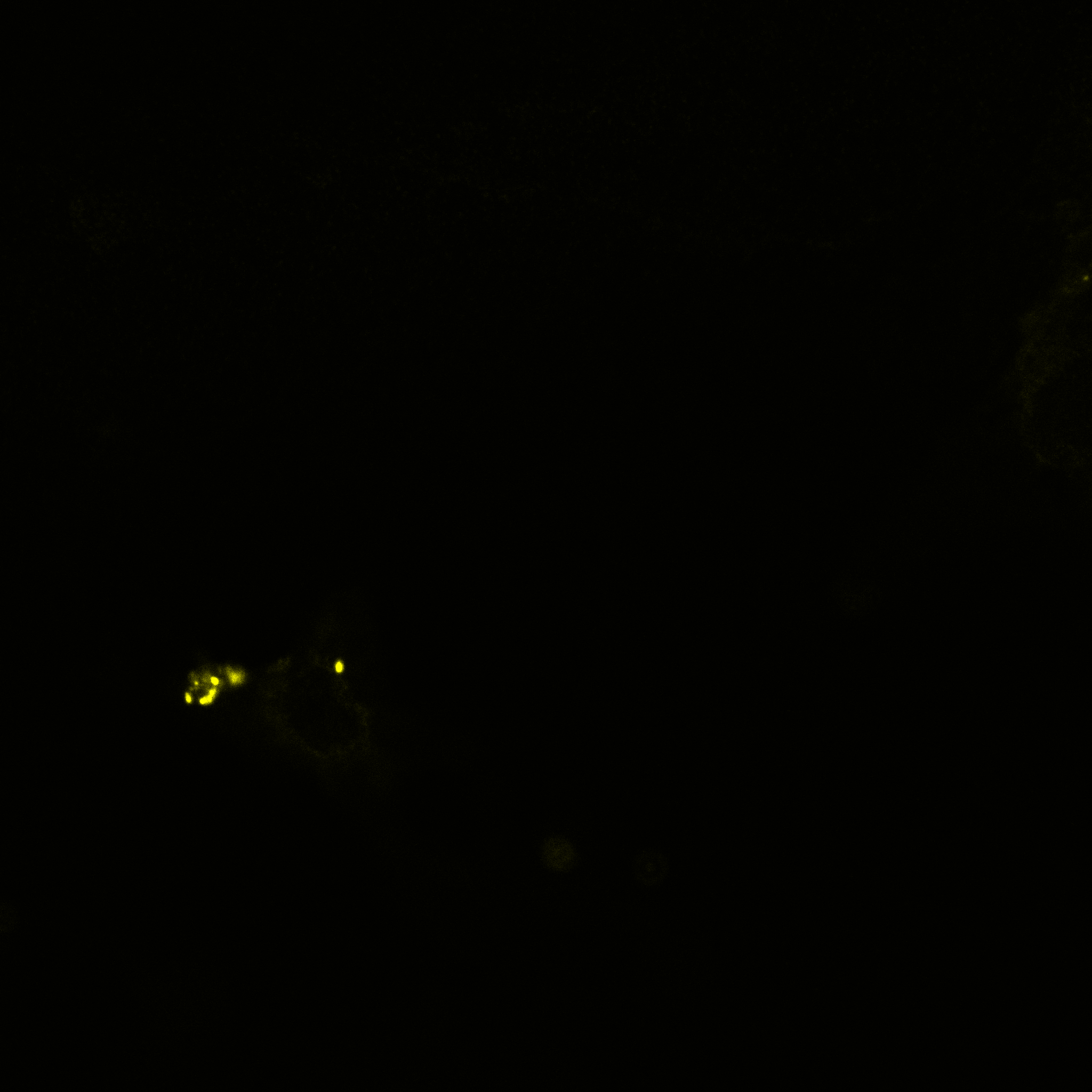

Supplement: Supplementary file 9 — Source Data for Figure 5 [file EMBJ-42-e111450-s005.zip › Source data Figure 5/5F&G/5F opsSRBC PKH26.gif]

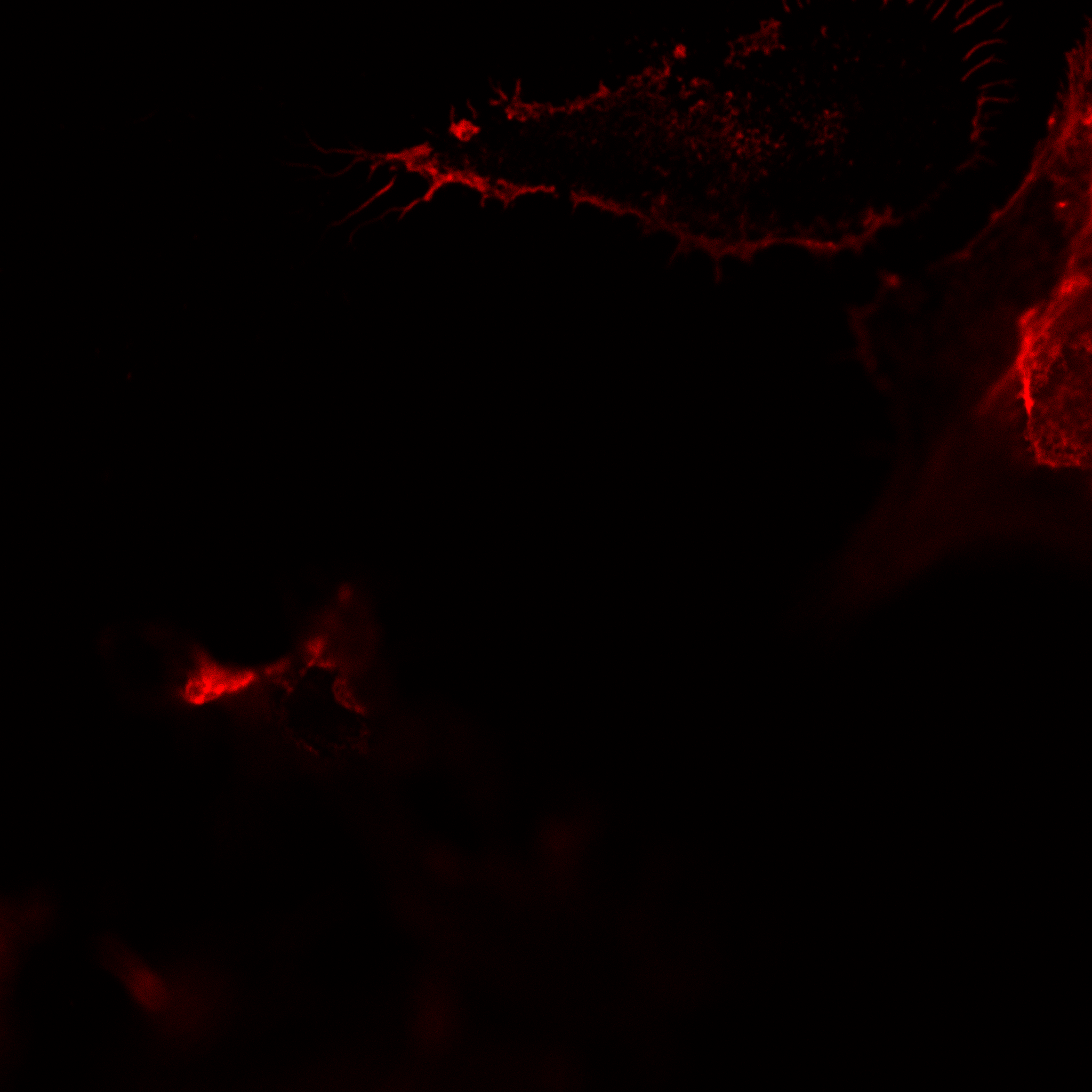

Supplement: Supplementary file 9 — Source Data for Figure 5 [file EMBJ-42-e111450-s005.zip › Source data Figure 5/5F&G/5F Phalloidin.gif]

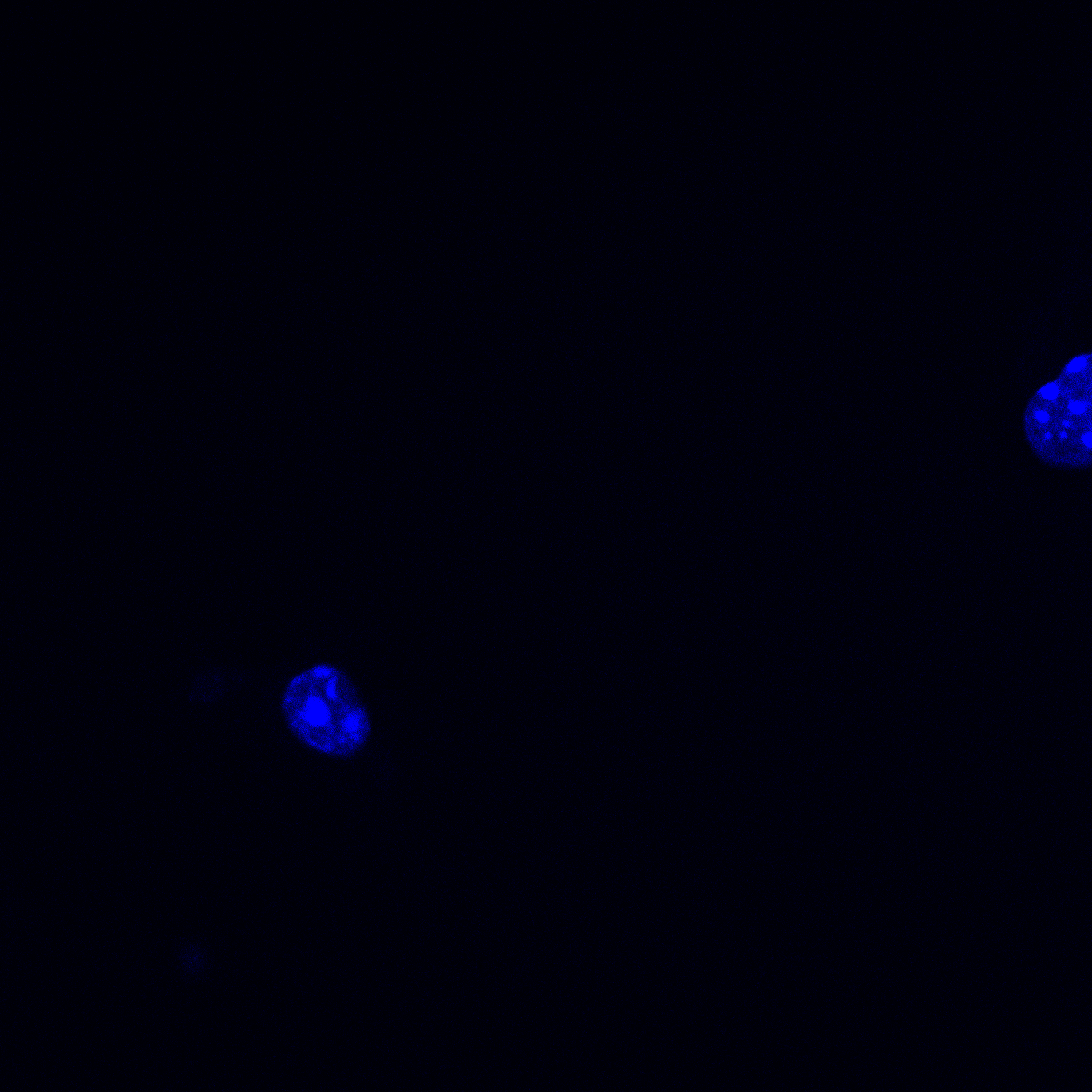

Supplement: Supplementary file 9 — Source Data for Figure 5 [file EMBJ-42-e111450-s005.zip › Source data Figure 5/5F&G/5F DAPI.gif]

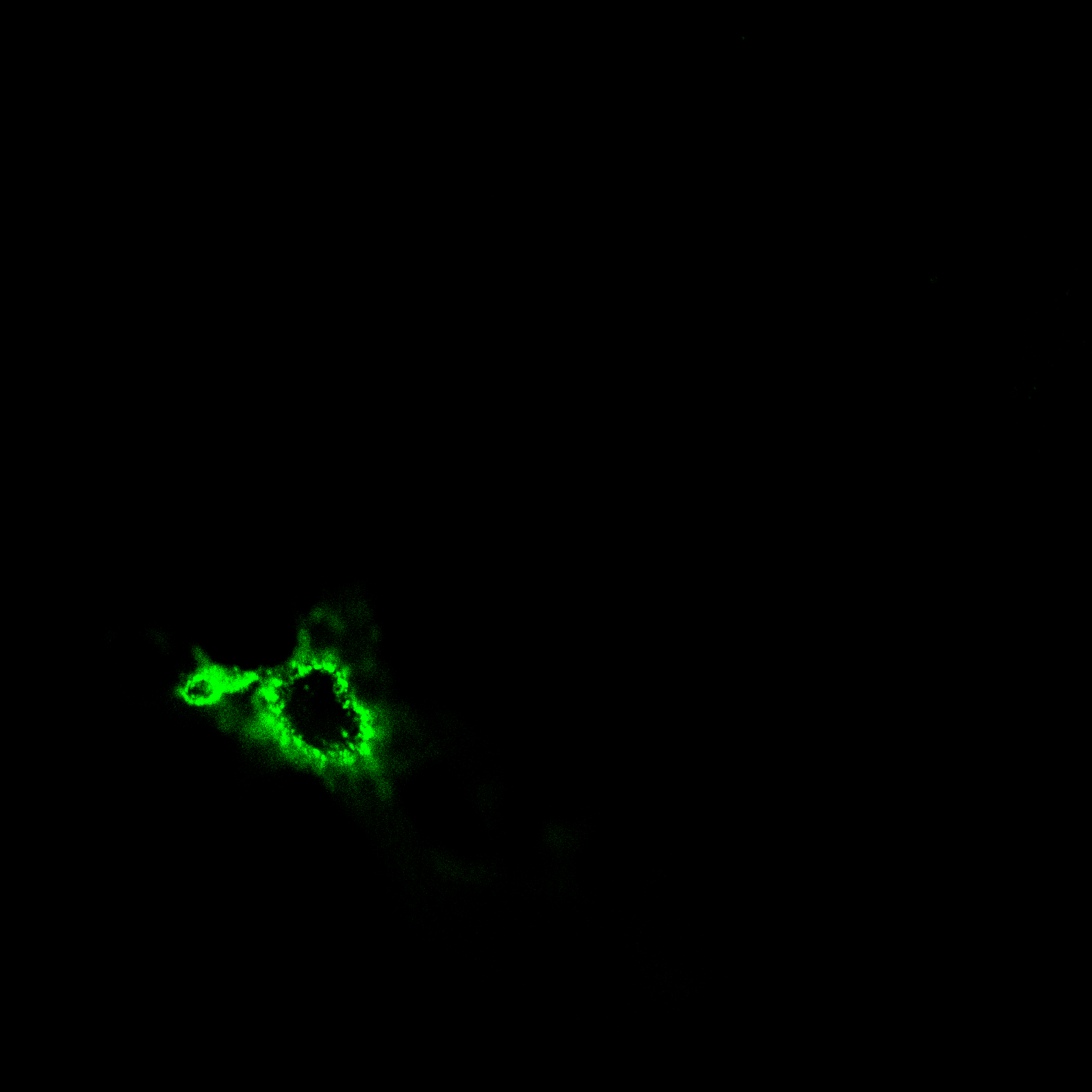

Supplement: Supplementary file 9 — Source Data for Figure 5 [file EMBJ-42-e111450-s005.zip › Source data Figure 5/5F&G/5F CALHM6-GFP.gif]

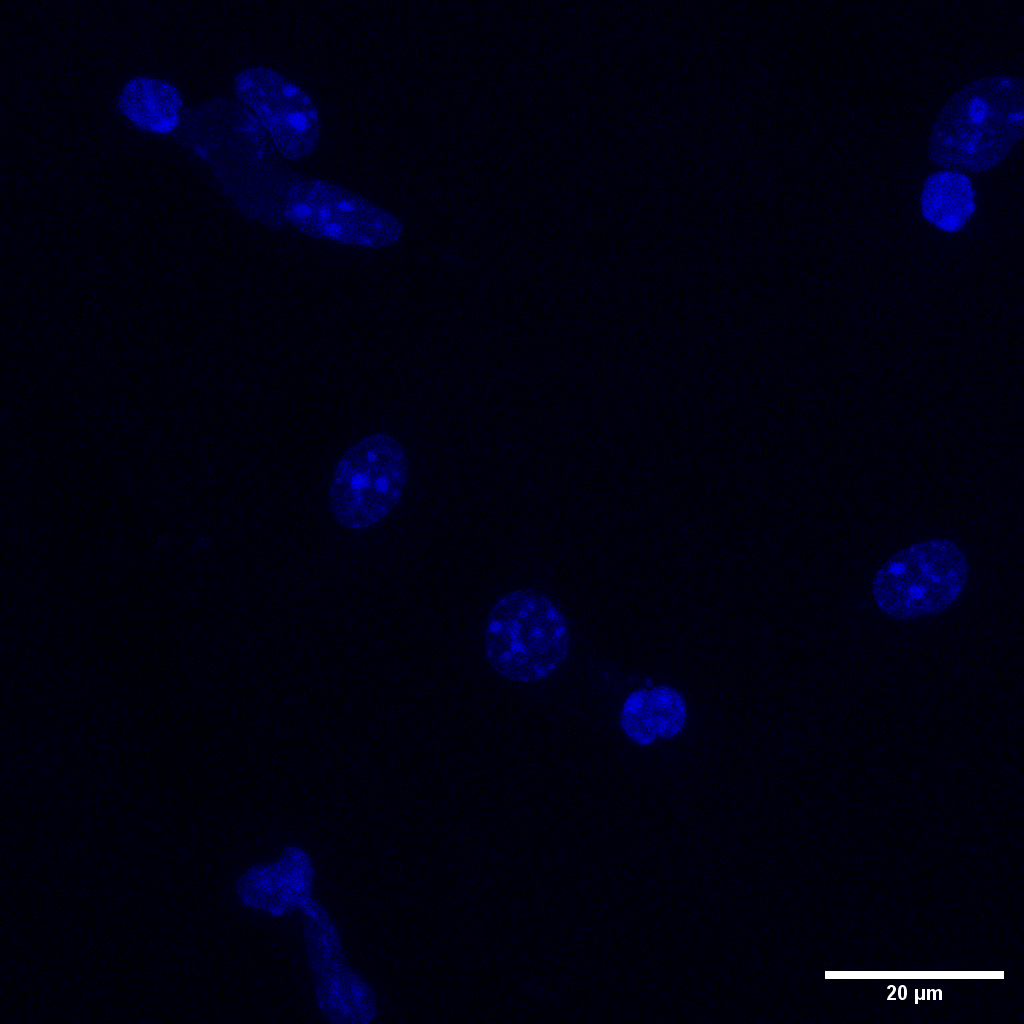

Supplement: Supplementary file 9 — Source Data for Figure 5 [file EMBJ-42-e111450-s005.zip › Source data Figure 5/5D&E/5D DAPI.gif]

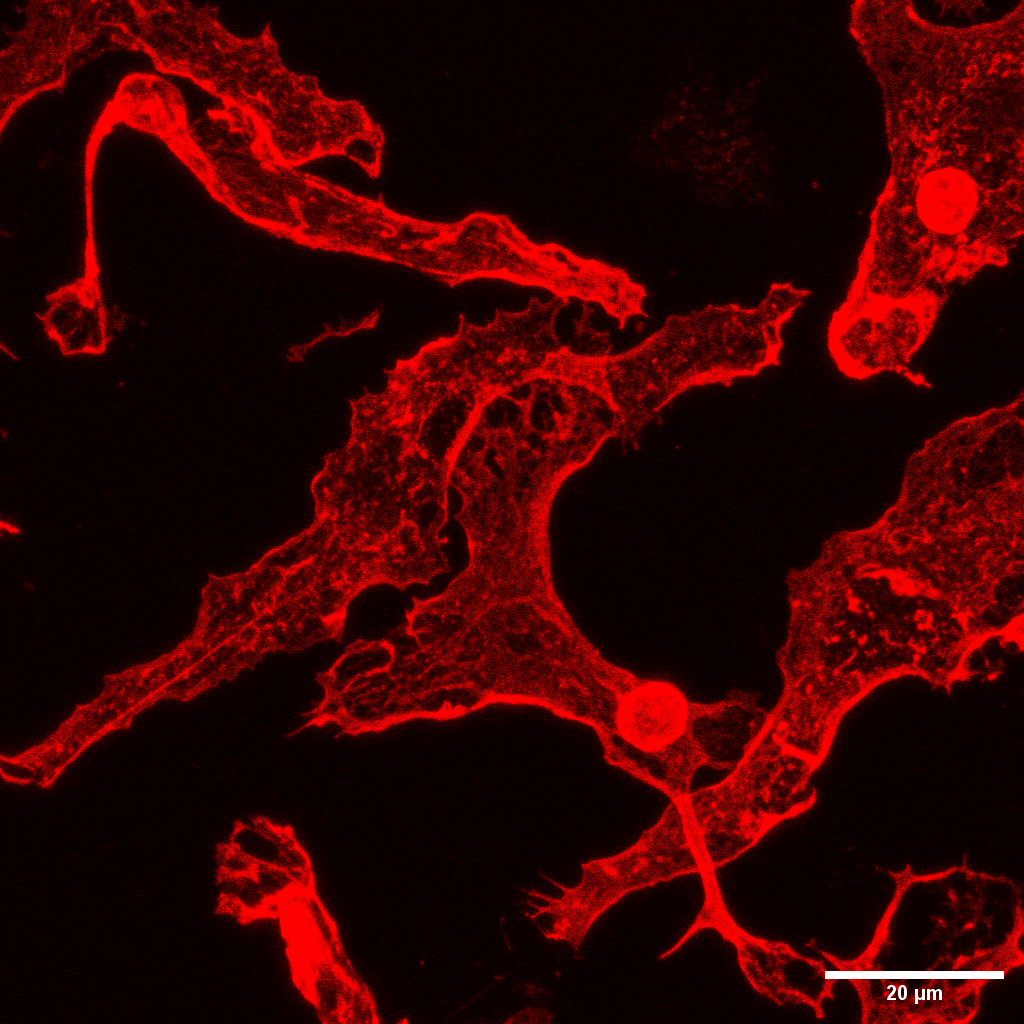

Supplement: Supplementary file 9 — Source Data for Figure 5 [file EMBJ-42-e111450-s005.zip › Source data Figure 5/5D&E/5D Phalloidin.gif]

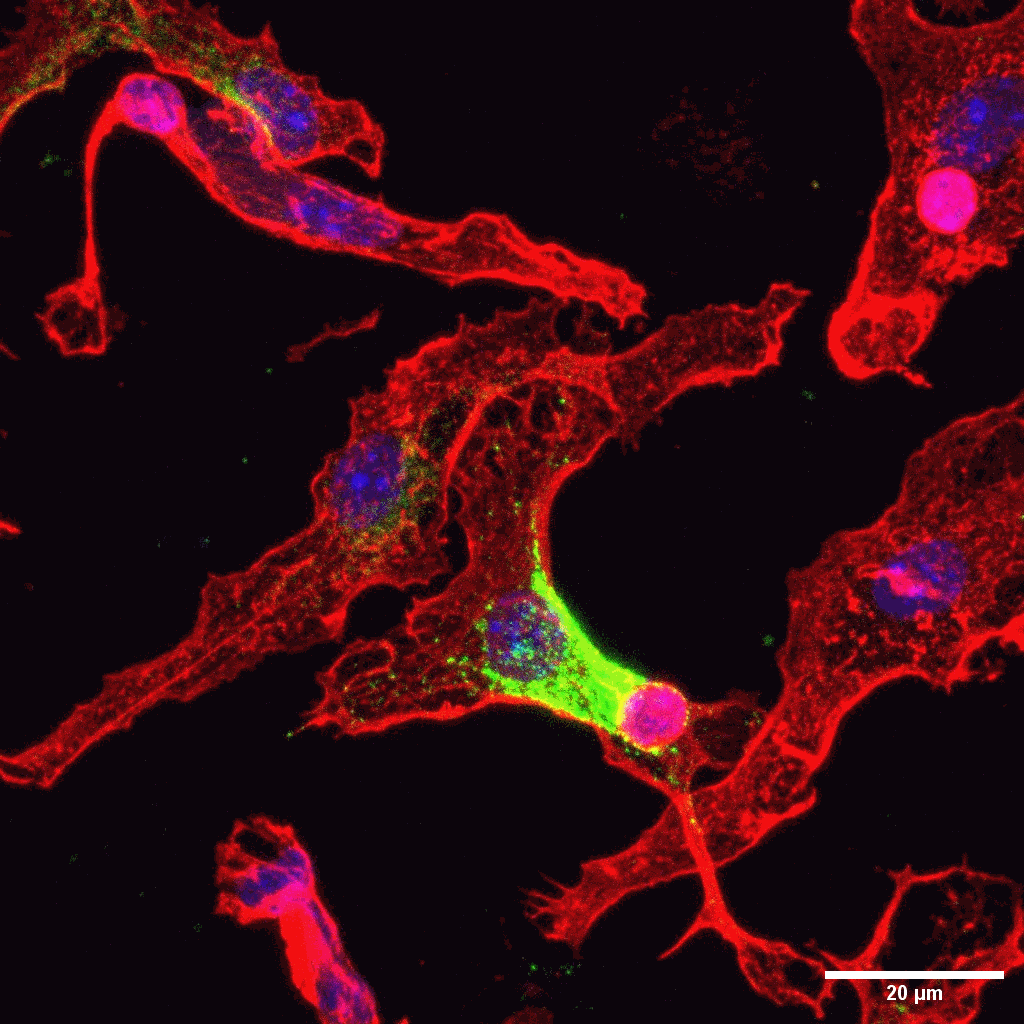

Supplement: Supplementary file 9 — Source Data for Figure 5 [file EMBJ-42-e111450-s005.zip › Source data Figure 5/5D&E/5D all colours.gif]

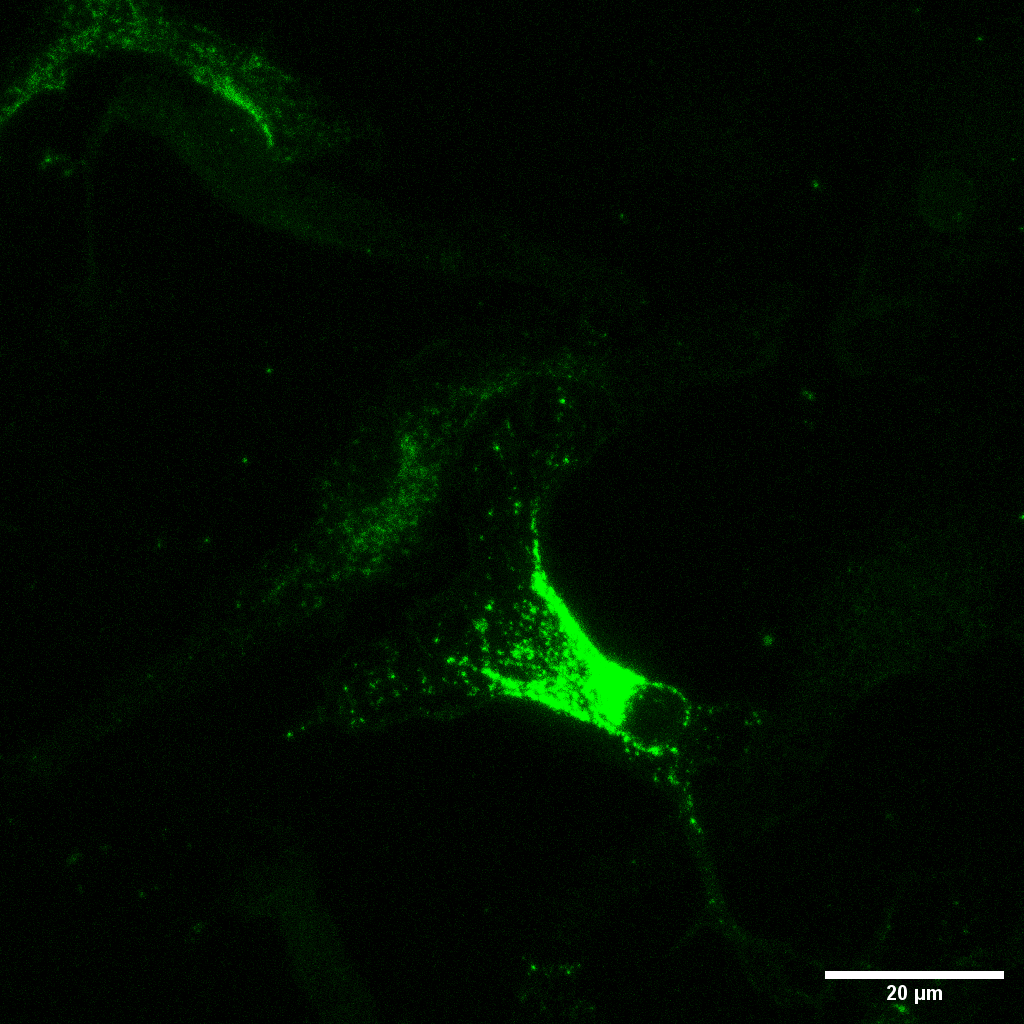

Supplement: Supplementary file 9 — Source Data for Figure 5 [file EMBJ-42-e111450-s005.zip › Source data Figure 5/5D&E/5D CALHM6-GFP.gif]

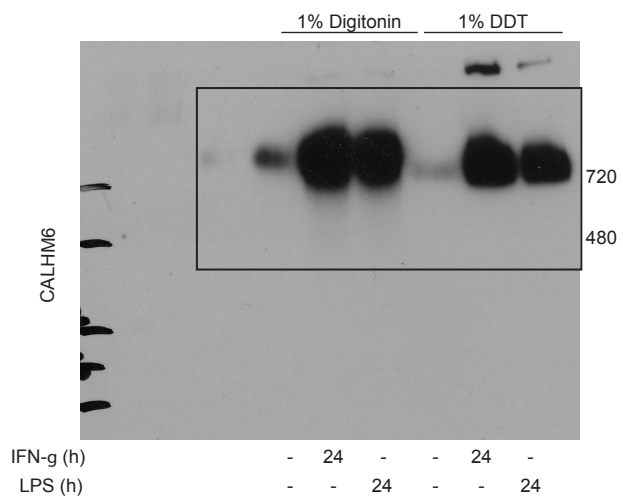

Supplement: Supplementary file 10 — Source Data for Figure 6 [file EMBJ-42-e111450-s011.zip › Source data Figure 6/6A/6A.pdf]

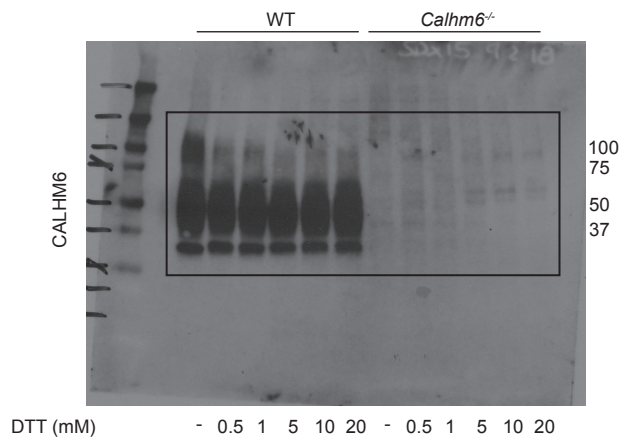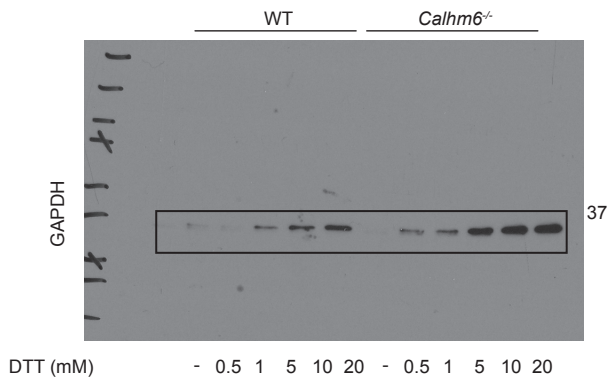

Supplement: Supplementary file 10 — Source Data for Figure 6 [file EMBJ-42-e111450-s011.zip › Source data Figure 6/6B/6B.pdf]

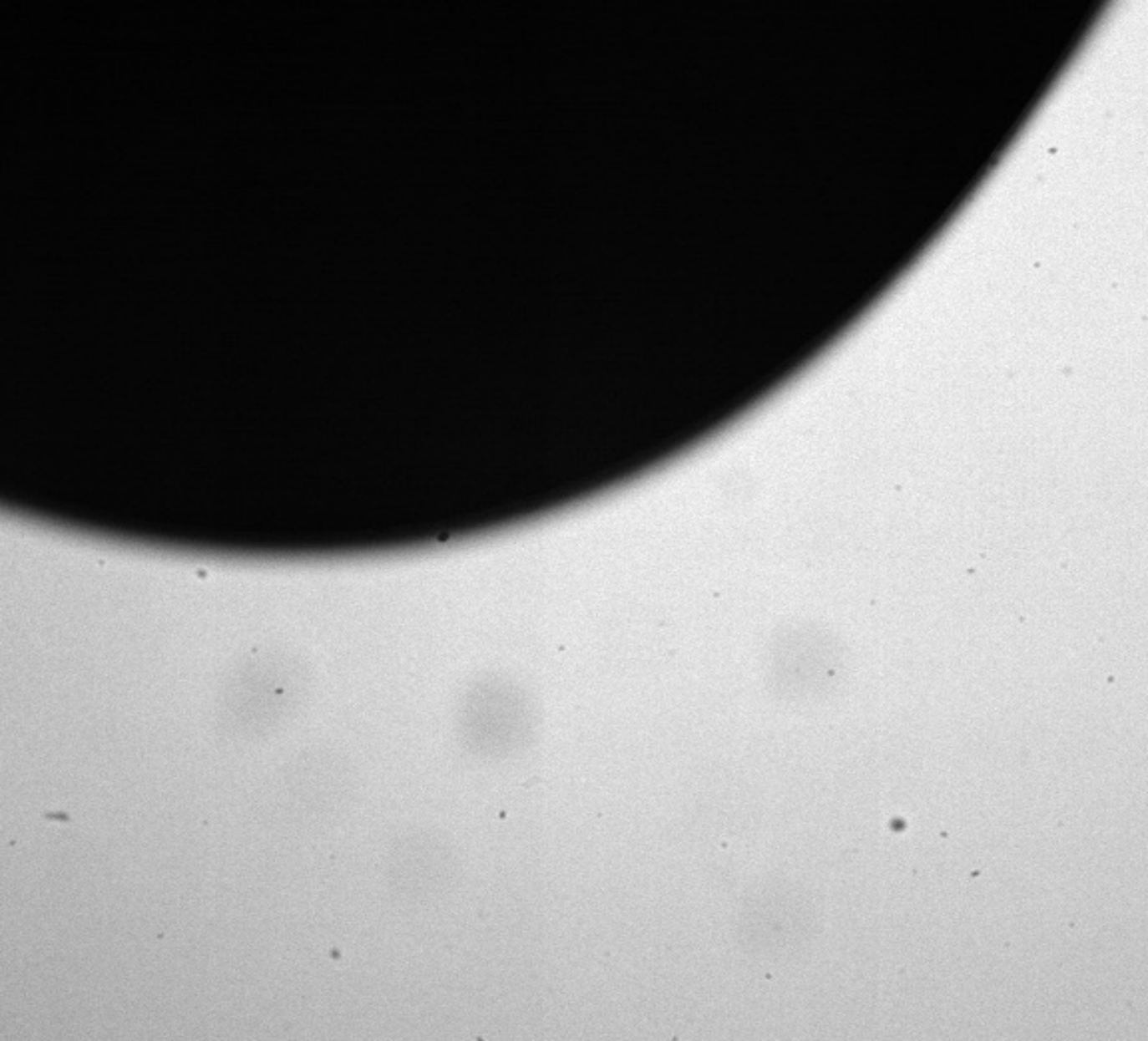

Supplement: Supplementary file 10 — Source Data for Figure 6 [file EMBJ-42-e111450-s011.zip › Source data Figure 6/6C/6C E119R CALHM6 GFP.pdf]

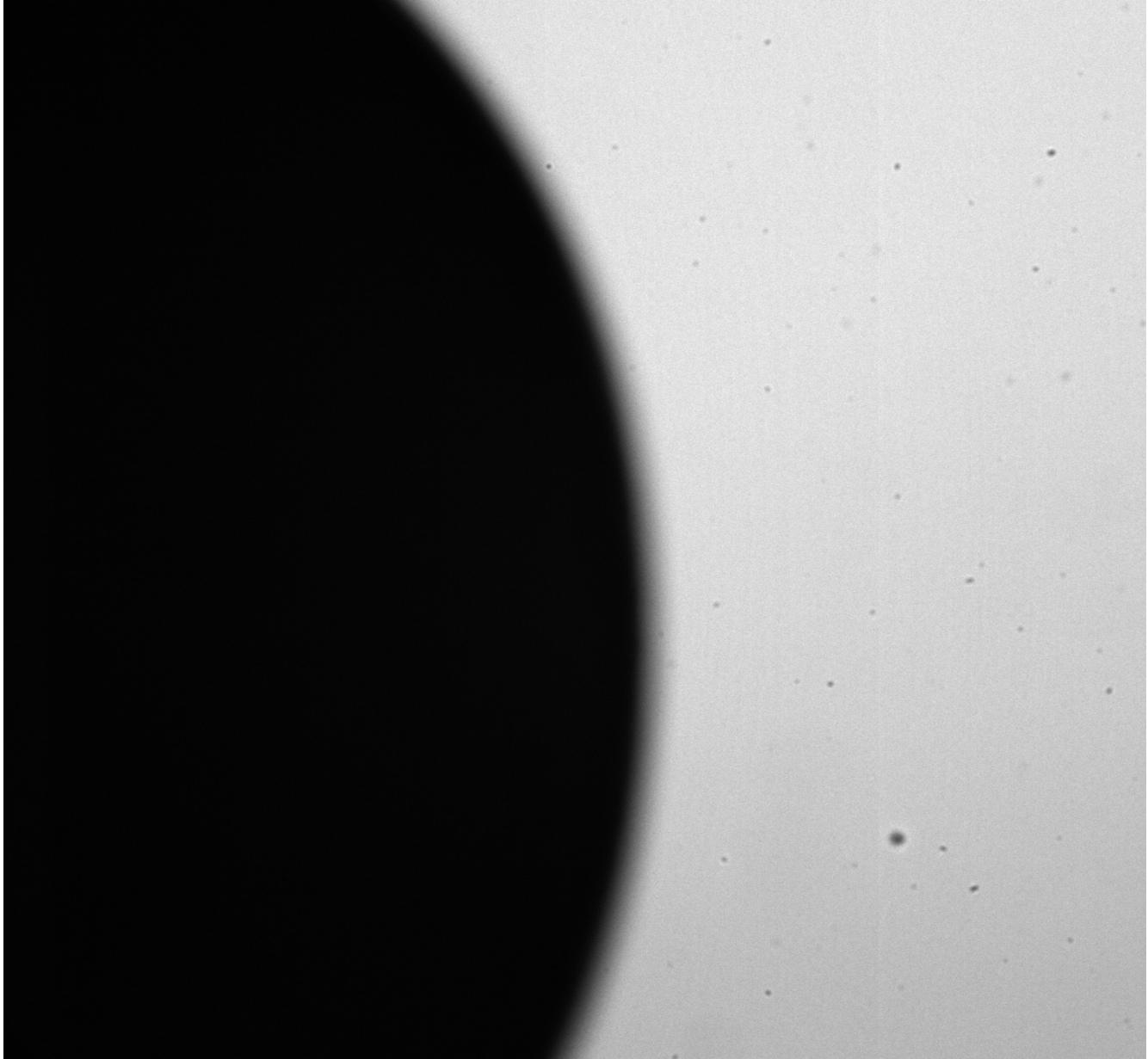

Supplement: Supplementary file 10 — Source Data for Figure 6 [file EMBJ-42-e111450-s011.zip › Source data Figure 6/6C/6C WT CALHM6 GFP.pdf]

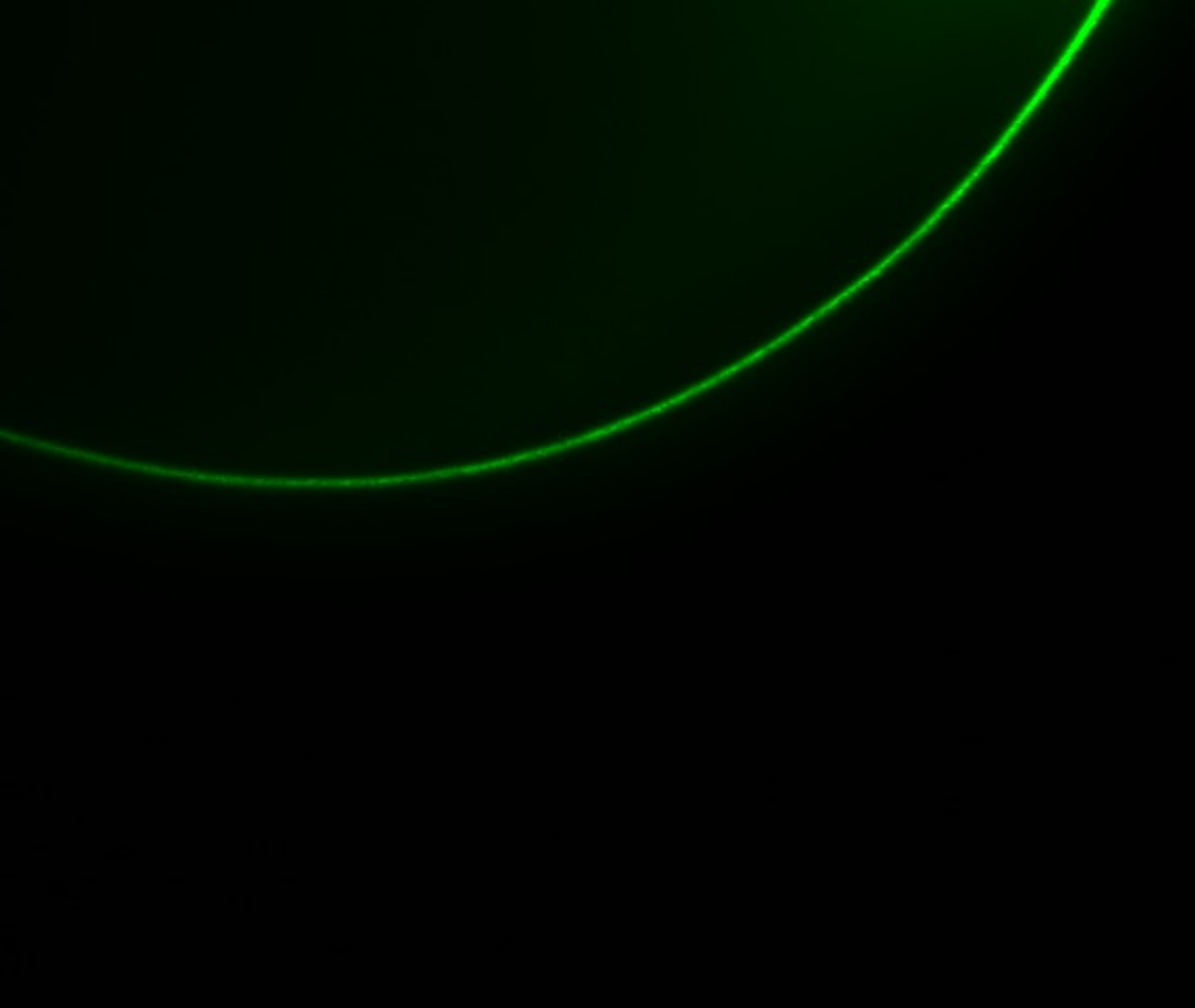

Supplement: Supplementary file 10 — Source Data for Figure 6 [file EMBJ-42-e111450-s011.zip › Source data Figure 6/6C/6C confocal E119R CALHM6 GFP.pdf]

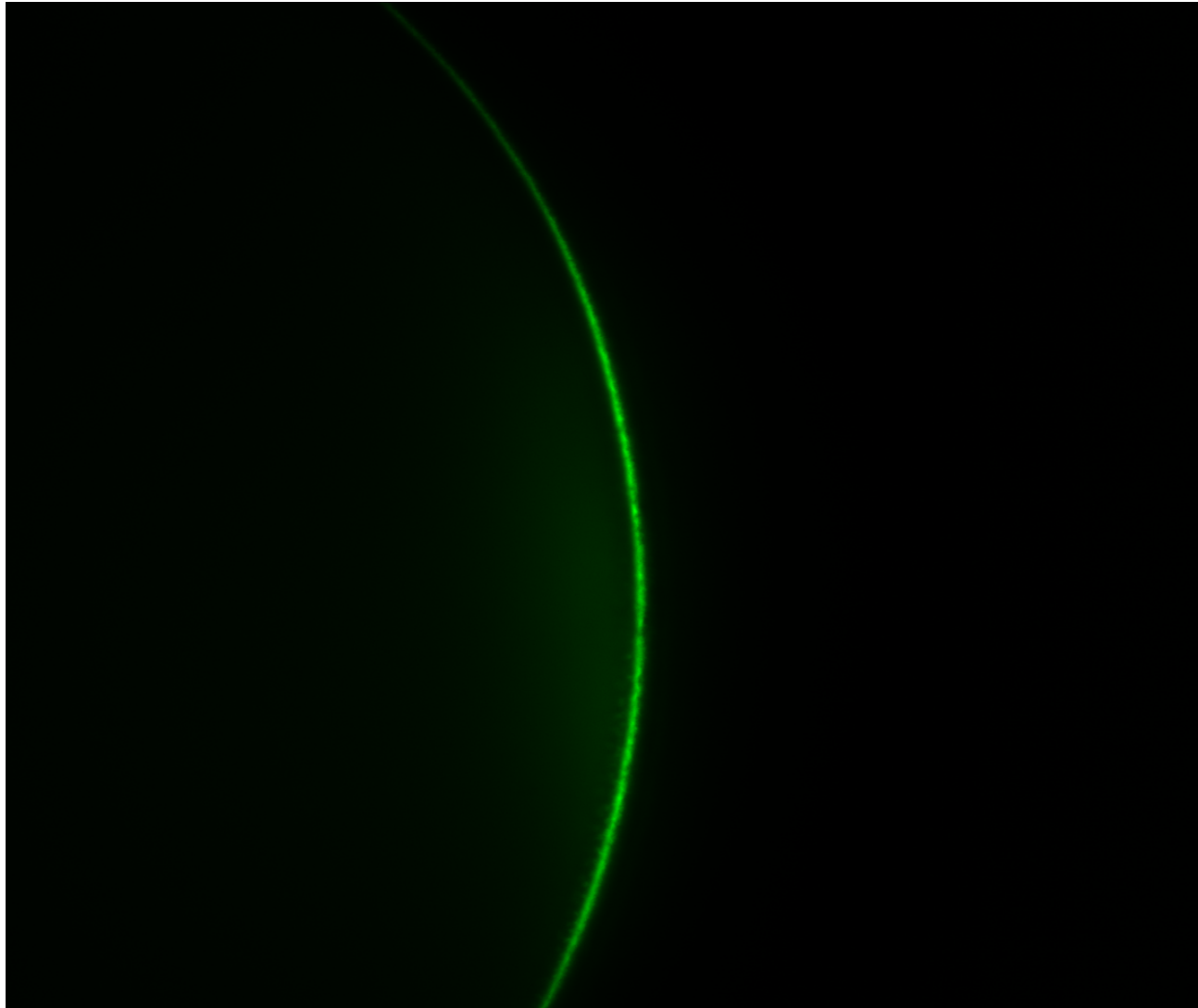

Supplement: Supplementary file 10 — Source Data for Figure 6 [file EMBJ-42-e111450-s011.zip › Source data Figure 6/6C/6C confocal WT CALHM6 GFP.pdf]

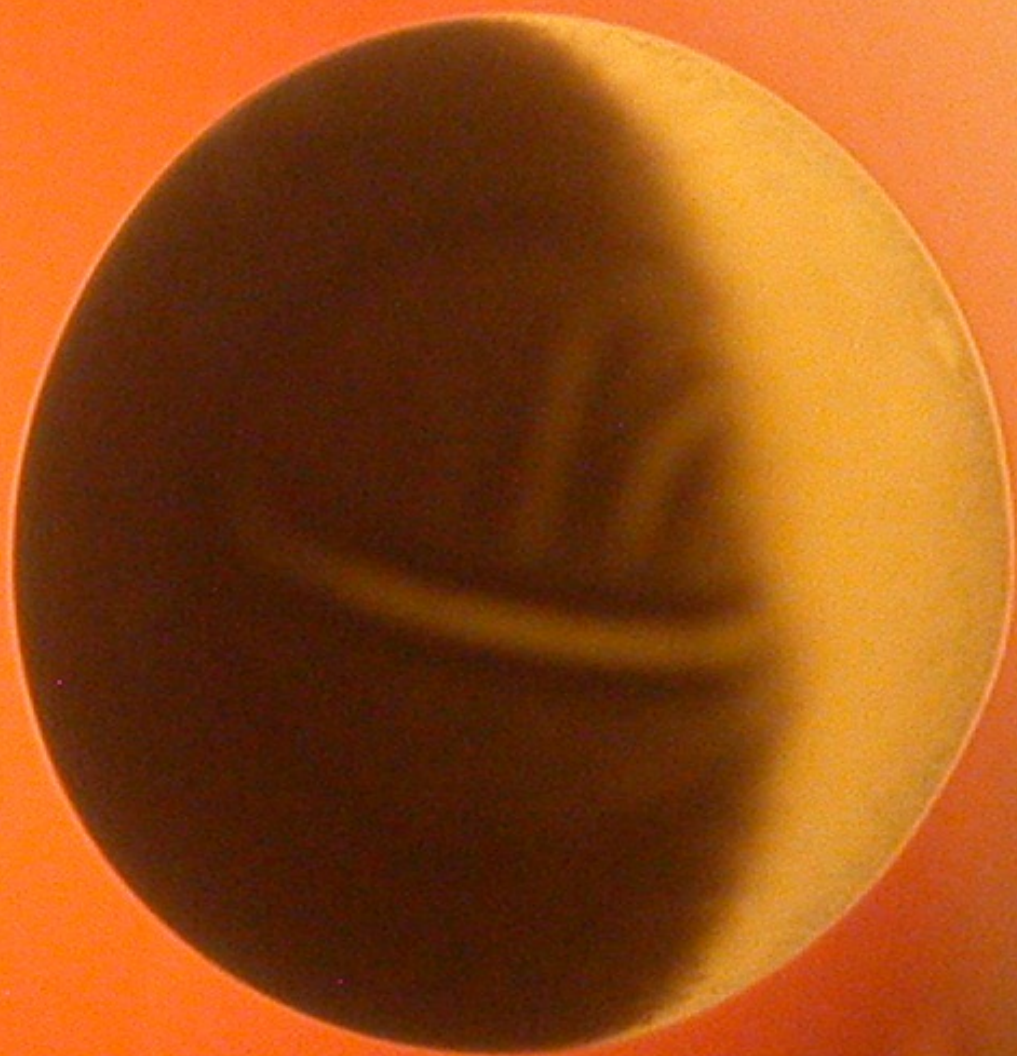

Supplement: Supplementary file 10 — Source Data for Figure 6 [file EMBJ-42-e111450-s011.zip › Source data Figure 6/6D/6D E119R-CALHM6.pdf]

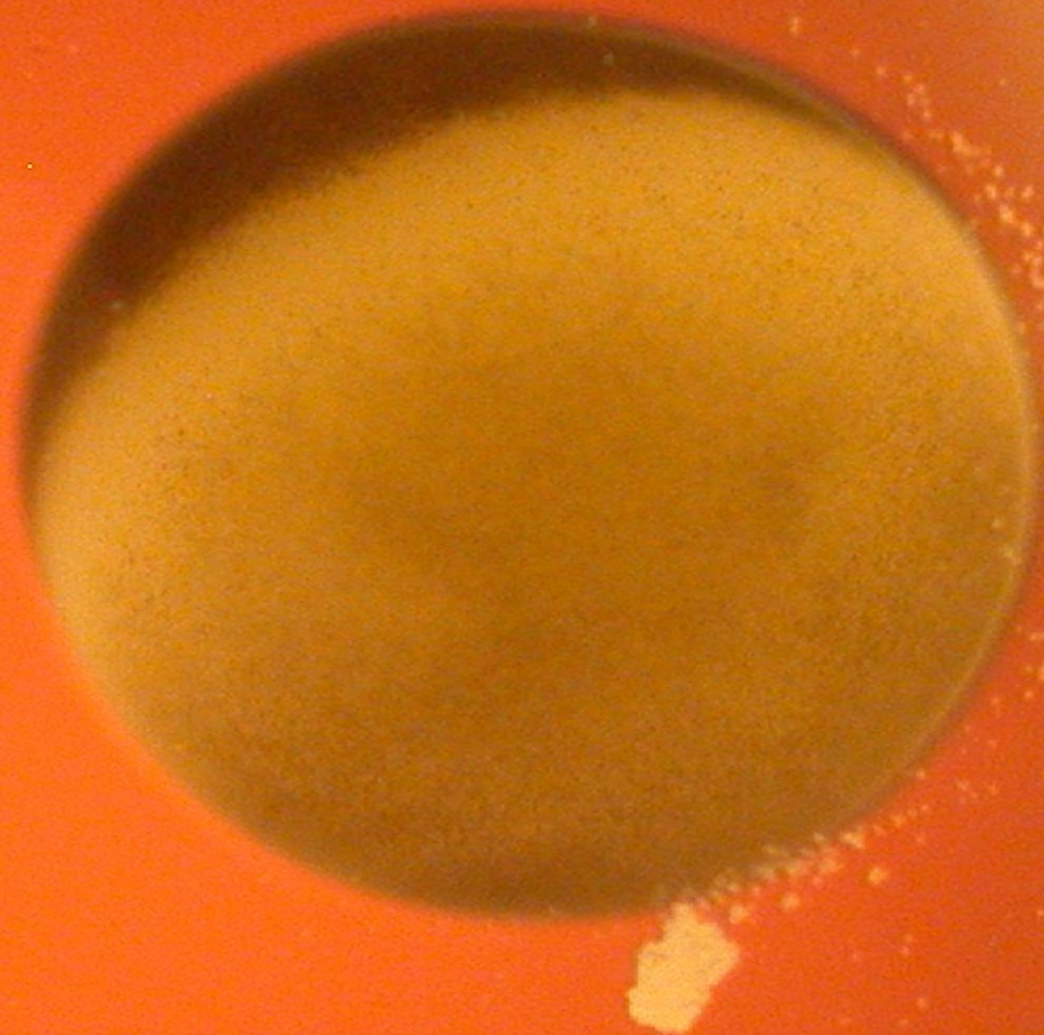

Supplement: Supplementary file 10 — Source Data for Figure 6 [file EMBJ-42-e111450-s011.zip › Source data Figure 6/6D/6D WT-CALHM6 5 ng.pdf]

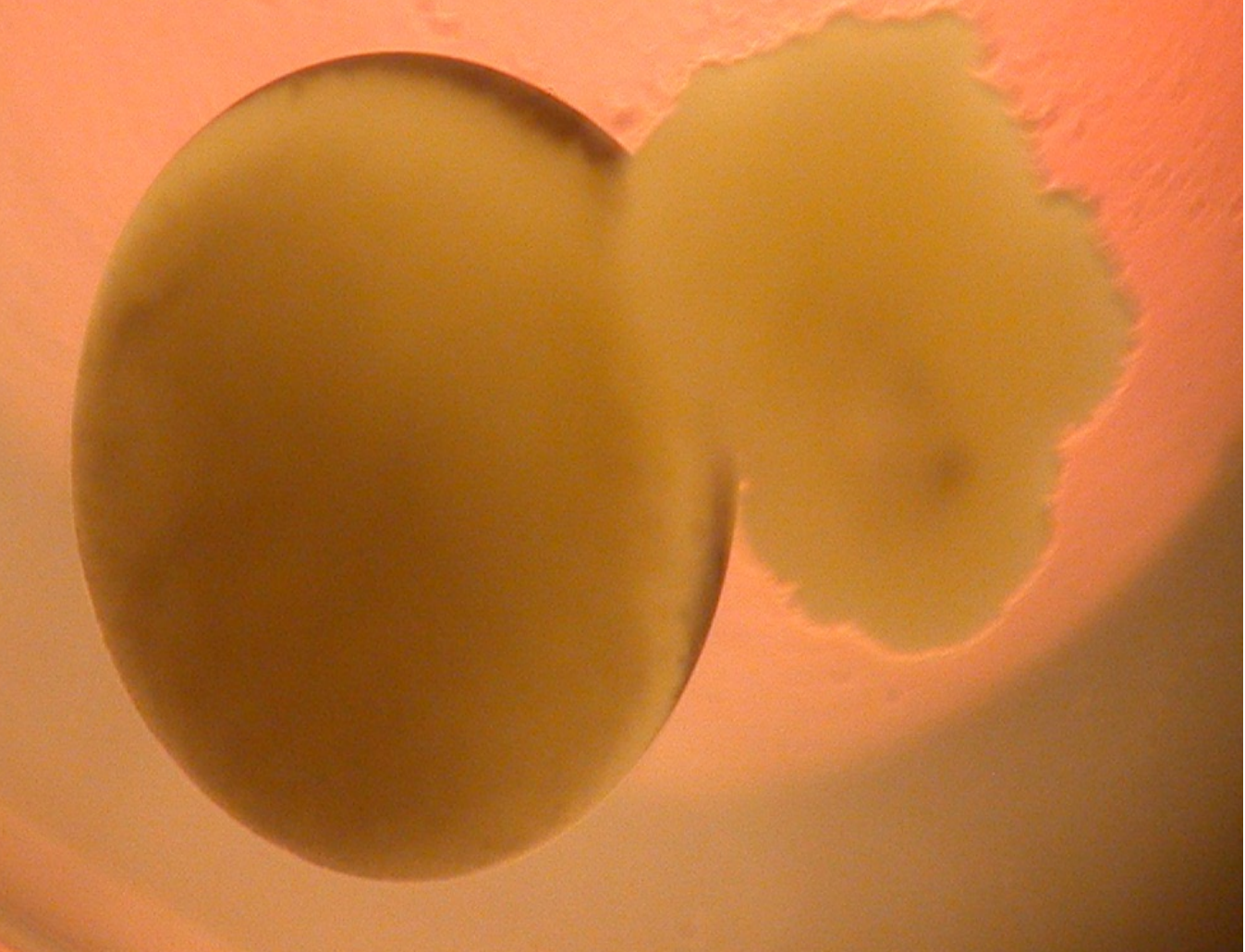

Supplement: Supplementary file 10 — Source Data for Figure 6 [file EMBJ-42-e111450-s011.zip › Source data Figure 6/6D/6D WT-CALHM6 50 ng.pdf]

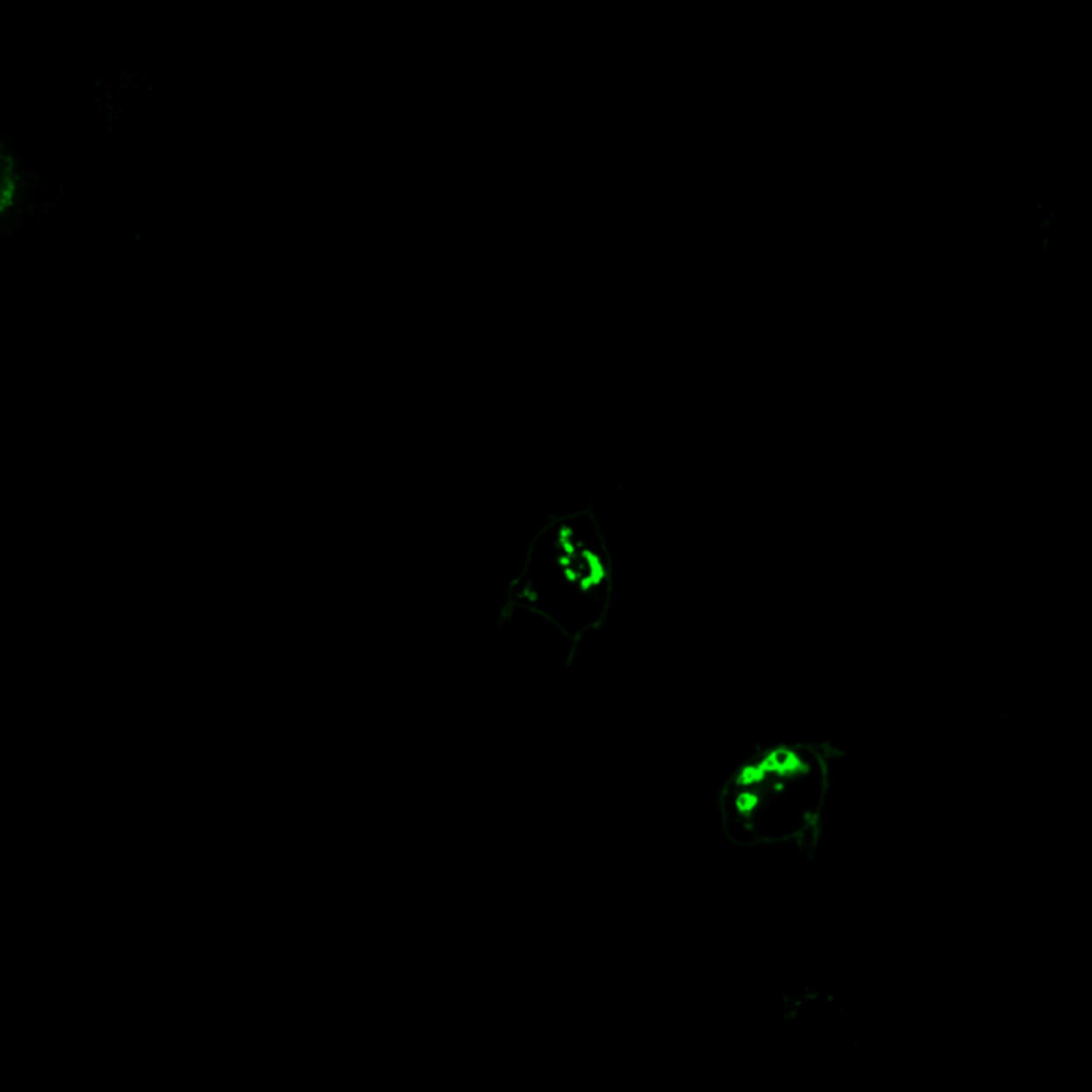

Supplement: Supplementary file 10 — Source Data for Figure 6 [file EMBJ-42-e111450-s011.zip › Source data Figure 6/6E/6E mCALHM6-GFP.pdf]
