# Supplementary material for: The ion channel CALHM6 controls bacterial infection‐induced cellular cross‐talk at the immunological synapse
Source: EMBO J. 2023 Mar 2;42(7):e111450. doi: 10.15252/embj.2022111450 (PMC10068325; doi:10.15252/embj.2022111450)
Supplement: Supplementary file 3 — Source Data for Expanded View [file EMBJ-42-e111450-s009.zip › Source_data_Expanded_view_Figure_1.zip!/Source data Expanded view Figure 1/EV1B/EV1B.pdf]

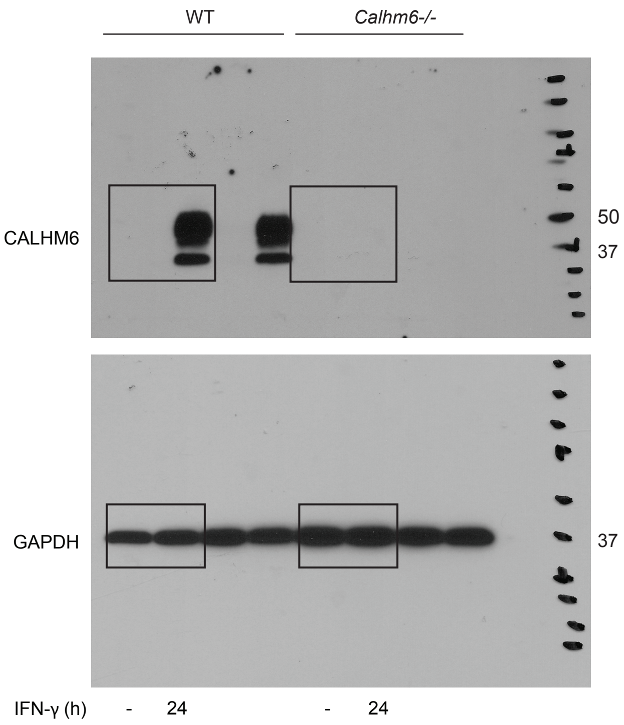
WT
Calhm6-/-
CALHM6
50
37
GAPDH
37
IFN-γ (h)
-
24
-
24
